# Speech and language pathologists' diagnostic approaches to developmental language disorders in German-speaking children with intellectual disability

**DOI:** 10.3389/fresc.2026.1925293

**Published:** 2026-09-22

**Authors:** Jenny Thillmann, Tina Ghaemi, Anna-Lena Scherger

**Affiliations:** Department of Rehabilitation Sciences, Research Unit of Language and Communication, TU Dortmund University, Dortmund, Germany

**Keywords:** cognitive impairment, developmental language disorder, diagnostics, intellectual disability, multilingualism, speech and language pathology, survey

## Abstract

**Introduction:**

Children with an intellectual disability (ID) often manifest a developmental language disorder (DLD). However, conventional standardized DLD diagnostic tools often prove unsuitable for this population. Furthermore, no guidelines are provided on how speech and language pathologists (SLPs) should conduct the assessment. This study investigated how practitioners in German-speaking countries assess DLD in children with ID, including the role of multilingualism and practitioners’ self-perceived competence.

**Methods:**

A cross-sectional online survey was conducted with 102 practitioners in German-speaking countries. The questionnaire examined diagnostic timing and goals, use of assessment tools, consideration of non-linguistic abilities, handling of norm values, adaptations for different levels of cognitive functioning, multilingual assessment practices, and perceived diagnostic competence regarding children with ID. Data were analyzed using descriptive statistics, qualitative content analysis for open responses, and multiple linear regression to identify predictors of self-perceived competence.

**Results:**

The Results showed that practitioners mainly use language assessment at the beginning of an intervention and for monitoring progress, with a focus on identifying specific language difficulties and planning intervention rather than categorical diagnosis. Most SLPs relied on tools developed for typically developing children, while adapting procedures for children with ID through observational and qualitative approaches. Non-linguistic factors such as attention, memory, and executive functions were frequently integrated into assessment. Norm-referenced testing was used variably, often for orientation rather than diagnosis. Multilingualism rarely led to different diagnostic procedures, although uncertainty among practitioners regarding its impact on language development was evident. Self-perceived competence was moderate overall and was significantly predicted by professional experience and working in specialized therapy settings.

**Discussion:**

In conclusion, assessment practices are highly heterogeneous and characterized by a combination of standardized tools and individualized adaptations. The findings highlight the need for more suitable diagnostic instruments, clearer guidelines, and targeted professional training to support consistent and confident DLD assessment of children with ID.

## 1 Introduction

In their daily operations with children, speech and language pathologists (SLPs) do not always encounter isolated clinical pictures like those covered by the literature. Children with developmental language disorder (DLD) often exhibit complex and heterogeneous profiles. They may also have comorbid disorders or underlying medical conditions that affect the child's language development. This, in turn, can potentially complicate diagnostic procedures. In addition, assessment of children who are raised in multilingual environments may present more complex diagnostic challenges for SLPs (1). Despite the expansion of research on DLD and its terminology (2), SLPs cannot always rely on the availability of assessment tools or evidence-based guidelines for diagnosing specific disorder profiles. However, a comprehensive and thorough evaluation of language development forms the basis for developing a therapy plan, which aims to facilitate children's ability to communicate their needs, engage in social interactions, partake in play, and share perspectives with their peers (3).

The question thus arises as how SLPs proceed in cases where there are no established guidelines (so- called gold standard) for assessing the language development of a child who, in addition to language developmental issues, has an underlying intellectual disability (ID). Which procedures are employed and how competent do SLPs feel in this field? The provision of answers to these questions could contribute to the identification of research gaps and needs, contributing to the improvement of diagnosis of children with DLD and an ID. To this end, an online-survey was conducted among SLPs and other professionals specialized in DLD diagnostics in German-speaking countries.

### 1.1 Terminology

For the present study, a comprehensive consideration of the definitions of DLD and of IQ-related diagnoses is necessary. The concerned terms are defined differently in various classification contexts. These contexts are, on the one hand, research and practice in speech and language pathology, which have undergone a development in the definition of DLD. On the other hand, there is a definition of DLD in the eleventh revision of the International Classification of Diseases (ICD-11) of the World Health Organization (WHO). ICD-11 is a global standard for recording health information and is used in medical contexts as a diagnostic system that is also relevant for billing medical services.

DLD is defined as a disorder of language development in childhood and adolescence in which productive and receptive language can be impaired in different linguistic-communicative domains (phonology, vocabulary, grammar, pragmatics, narration, and verbal working memory). Most of those domains can be tested by means of standardized tests. The various definitions agree on this (1, 4, 5).

However, the named definitions differ slightly in terms of how DLD relates to other cognitive disorders. The term ID plays a significant role in this context.

ICD-11 and the American Association on Intellectual and Developmental Disabilities (AAIDD) define disorders of intellectual development (ICD-11 code 6A00) as a group of etiologically diverse conditions originating during the developmental period up to the age of 18. The main characteristics for an ID are, on the one hand, a below average intellectual functioning, which affects reasoning, planning, problem solving, abstract thinking, understanding complex ideas, rapid learning, and learning from experience. On the other hand, adaptive behavior is affected, which includes conceptual, social, and practical everyday activities (6). Below average is hereby defined as scoring two standard deviations below the mean in an intelligence test. This clearly addresses below-average IQs of < 70 (5).

The IQ range between 70 and 85 is not listed as a specific category in the ICD-11. Internationally, this range is often referred to as borderline intellectual functioning (BIF) but not classified as a disability (7). In Germany, the IQ between 70 and 85 was long referred to as a “learning disability” (8–10) and was therefore pathologized to a greater extent compared to the international standard. Coming to definitions of DLD and their connections and distinctions from cognitive impairments, research and practice have long been guided by the definition of “specific language impairment” (SLI), which explicitly excluded children with a low IQ and set the limit at > 85. According to this definition, SLI was limited to an isolated disorder in understanding and/or producing spoken language. A low IQ was excluded in order to minimize the influence of cognitive factors on language performance (11, 12) Following many decades of discussion, a study was conducted to reach a consensus on uniform terminology among experts in language acquisition in both English-speaking and German-speaking countries. As part of the CATALISE consensus study in English-speaking countries, experts in language acquisition agreed on a replacement of SLI by the more comprehensive term DLD (2). The new definition is not based on exclusion criteria. Instead, it is based on a threefold distinction between risk factors, co-occurring conditions and differentiating conditions. The latter are especially related to this study. A biomedical differentiating condition is a condition in which language disorder occurs as part of a more complex pattern of impairments. It should be referred to “language disorder associated with X”, where X stands for the differentiating condition. Differentiating conditions include among others genetic conditions such as genetic syndromes, autism spectrum disorder (ASD) and/or ID. It should also be considered that there may be overlap between these different conditions. For example, Down syndrome, ASD, and ID may be present simultaneously (3, 13).

It is important to note that, by means of the CATALISE consensus children with a low level of nonverbal ability (IQ 70–85) are also covered by the diagnosis of DLD and not by “language disorder associated with X”.

In the DELPHI consensus study for the German-speaking DACH countries (Germany, Austria and Switzerland) by Lüke et al. (4), experts agreed on the term “Sprachentwicklungsstörung = developmental language disorder” (SES = DLD). In addition to terminology, the German DELPHI study is consistent with the CATALISE study in most points. Also, in the German-speaking community a consensus has been reached that children with a primary underlying condition, such as ID (non-verbal IQ < 70), ASD, or genetic syndromes, should be classified as having “developmental language disorder associated with X” (DLD associated with X, short: DLD with X). However, the German study differs fundamentally in one respect: For less substantial difficulties in nonverbal intelligence (IQ 70–85), no consensus has been reached in German-speaking countries as to whether these children should be classified as having DLD or DLD with X (4). This means that children with an IQ between 70 and 85 can be covered by both terms.

The ICD-11 is in line with the DLD criteria of the two consensus studies (5). With regard to the origin of DLD, the ICD-11 states that DLD cannot be explained by other neurodevelopmental disorders, but that both can coexist. If the diagnostic criteria for an ID are met, and if language skills are significantly below what would be expected based on the individual's general intellectual functioning and adaptive behavior, then both diagnoses may be given (5). The severity of DLD in children with ID varies depending on their level of intelligence and is also dependent on any comorbidity that may be present (14, 15). For example, language development of children with ASD is not always impaired, whereas communicative development is altered in a characteristic way. In contrast, children with Down syndrome primarily exhibit receptive and expressive morphosyntactic disorders (16).

In the following, we refer to the DLD term that has been agreed upon in German-speaking countries: “DLD with X”, which can include both children with ID (IQ < 70) and children in the “BIF” IQ-range of 70–85). Consequently, for the purpose of this study, it has been determined that the population of interest encompasses both children with ID (IQ < 70) and children with cognitive functioning in the IQ range of 70–85). These groups are diagnostically distinct and are not considered equivalent. They were nevertheless considered jointly because both may present with cognitive difficulties relevant to language assessment. Furthermore, a clear distinction between the two may not always be feasible at the time of assessment.

### 1.2 Prevalence

Many prevalence studies were conducted prior to the terminology changes and consequently report a prevalence rate of approximately 7% for SLI/isolated DLD (17–19). Norbury et al. (20) obtained similar figures in England for speech and language disorders of “unclear origin” (7.58%). This includes children with BIF, but not those who have an associated biomedical condition. The prevalence of DLD with ID or another medical diagnosis was 2.34%, summing up to a total of 9.92% (20).

DLD prevalence data from Germany is not available. However, some reports published by German insurance companies state that 11.5% of all children received speech therapy where DLD is the main reason for such therapy (21). Further information on diagnostic criteria and associated biomedical conditions is not available. Another health insurance company recorded an increase in diagnoses of “specific developmental disorders of speech and language” (F.80), rising from 10.7% to 13.2% between 2010 and 2021 (22).

Prevalence for ID range from one to two percent of all children and adolescents, depending on the disorders included in the calculations (23–26).

Given variation in terminology across countries, the comparability and validity of reported prevalence remains unclear.

### 1.3 International language diagnostics for children with ID

Even though recent studies have moved away from condition-specific language interventions for children with ID and advocate a transdiagnostic perspective in the planning and delivery of interventions (27), previous diagnostic tools have not been developed comprehensively for language assessment in children with ID. Instead, the characteristics of individual genetic syndromes or other biomedical conditions have been taken into account in the diagnostic tools.

Since Down syndrome is the most common genetic cause of ID (3) and is the subject of much research, diagnostic tools for this condition are presented as examples here. One is the Language Assessment and Training Support System (LATSS) software system which was developed specifically for children with Down syndrome under the age of 6. It combines assessment and training in a customized program, but is not a standardized, widely used clinical tool (28). A Spanish version of the MacArthur-Bates Communicative Development Inventories, which are widely used to study early language and communicative development, has been created for children with Down syndrome (CDI-Down). It refers to the disorder-specific linguistic and communicative profile of these children and demonstrates good validity and reliability (29). Thurman et al. (30) developed expressive language benchmarks for children with Down syndrome for phonology, vocabulary, and grammar. This classification into milestones is intended to serve as a guide to the children's language abilities and contribute to the identification of treatment goals. These are just a few examples of international tests that relate to the language development of children with Down syndrome.

Due to the high degree of individuality of ID and the many possible underlying medical conditions, there is no standardized diagnostic procedure exclusively for DLD with ID.

### 1.4 German language diagnostics for children with ID

Since language diagnostics are highly language-dependent, the following section focusses on German diagnostic tools, guidelines, and recommendations.

In Germany, recommendations for the diagnostic procedures of language development are provided in an AWMF-S2k guideline (“consensus-based guideline”) issued by the Arbeitsgemeinschaft der Wissenschaftlichen Medizinischen Fachangestellten (AWMF; Association of the Scientific Medical Societies in Germany) using outdated terminology (31). A revised version is planned for December 31, 2026. It will presumably include the updated DLD terms (4) with new diagnostic approaches.

Furthermore, there is an AWMF-S3 guideline of the German Society of Phoniatrics and Pediatric Audiology (Deutsche Gesellschaft für Phoniatrie und Pädaudiologie, DGPP) on the treatment of DLD (32), which also includes a chapter on the treatment of DLD with ID (33), (pp. 161–166). Since this guideline does not address diagnostics, practitioners are not provided with specific test recommendations or operationalized diagnostic cut-offs. However, with a focus on treatment, fundamental principles and goals are defined on which treatment should be based on (32). For children with ID, DLD diagnostics should also cover the linguistic-communicative aspects: phonological, semantic-lexical, morphological-syntactic, and pragmatic abilities, exactly the same as for DLD without any associated conditions. From early childhood onwards, verbal and non-verbal aspects should both be considered. Verbal aspects include expanding the child's vocabulary and the variety of sentence patterns they understand and produce, improving their intelligibility and phonological awareness, and developing their conversational skills. Nonverbal goals include improving nonverbal interaction, training shared attention, and the use of sign language to accompany speech. However, for children with ID the overall development plays a greater role. In addition to linguistic abilities, speech therapy should consider individual factors such as biomedical diagnosis, developmental stage, and awareness of the disorder and set those into relation to the linguistic abilities (32). Regarding the goals of the intervention, the guideline clearly states that improving communicative and linguistic abilities, and promoting or restoring social participation, are key objectives for children with DLD. For children with ID, early, developmentally appropriate and communication-centered intervention in a multidisciplinary setting is recommended (32).

In line with this, the goals of expanding vocabulary, promoting conceptual language comprehension, increasing sentence variety and complexity, promoting phonological awareness, and improved pragmatic skills are also reported internationally. Therapy planning should be based on the individual needs of the child, which are assessed using standardized testing procedures, parental assessments, and feedback from teachers (34).

To diagnose a DLD, the regular procedure is to use a standardized test in addition to observations and anamnestic interviews of parents and caregivers. Standardized tools offer various advantages over informal assessment (e.g., observation of spontaneous speech). With standardized tools performance can be assessed quantitatively, but qualitative criteria can also be evaluated using these tools. Specific target structures can be evoked, and language proficiency can be reliably measured and compared with the standard values of other children (35, 36). Neitzel & Dittmann (37) describe that, due to the large interindividual heterogeneity of the linguistic performance of children with Down syndrome, the development of standardized testing procedures, including age norms, seems hardly possible. A more extensive degree of heterogeneity becomes apparent upon consideration of all children with ID, with or without underlying syndromes.

A potential solution is the adaptation of standardized DLD diagnostic procedures in order to enable an appropriate diagnosis (37, 38).

Aktas (38) developed a theoretical framework model for development-oriented language assessment and support for children with ID in Germany. She discusses the level of ability at which a child starts with a particular tool and goes through a procedure consisting of various tests until a comprehensive picture of language reception and production has been established. The adapted test procedures are included in Aktas’ model: SETK-2 (39), SETK 3-5 (40), and the parent questionnaires ELFRA-1 and ELFRA-2 (41). For the evaluation, she presents an approach for interpreting the standard values differently, using them for orientation only. She furthermore presents a procedure for conducting a qualitative, theory-based evaluation of the test situation as a “standardized observation situation”.

Neitzel & Dittmann (37) also discuss various possibilities for test adaptations for children with Down syndrome, which can also be very relevant for children with ID. To this end, they review individual cases and discuss the applicability of the tests: TROG-D (42), AWST-R (43), and ESGRAF 4-8 (44). In their opinion, possible adaptations would be a) using mental age instead of chronological age for norm orientation in line with (45, 46), with special considerations for the interpretation and possible qualitative evaluation of the results. b) adapting the material to the child's level of development, reducing stimuli, and making adjustments for additional impairments, e.g., visual acuity/motor skills. c) Possible simplification of the instructions, division of the session into several appointments, and possible selection of specific subtests instead of entire test batteries (37).

### 1.5 DLD, ID and multilingualism

Monolingual acquisition is very heterogeneous and individual in children with ID and this variability is exacerbated in the context of multilingualism (47). In the present study, multilingualism is understood as the ability to communicate in two or more languages and to be regularly exposed to them, regardless of proficiency level, language modality (spoken/signed), or context (home, school, society) (48, 49). Language diagnostic practice faces further challenges, as there is a lack of adequate DLD diagnostic tools for multilingual children, and the test results of these children cannot be used with existing norm values (for monolingual children) (50). Research on multilingualism in children with cognitive developmental impairments usually focuses on the relationship between multilingualism and DLD. Increasingly, multilingual children with Down syndrome or ASD are also becoming the focus of attention (51–53).

The question arises as to whether multilingualism has an influence on language development in children with ID and whether this means that other adjustments may be necessary in diagnosis within this particular population. According to Ostad (54), it can be assumed that children with cognitive impairments are, in general, capable of acquiring more than one language. Studies with children with ID and Down syndrome show that bilingualism does not cause additional disorders in receptive and expressive language. They develop language skills in their second language in line with their development and similarly to bilingual comparison groups without Down syndrome (53, 55) It is assumed that effective acquisition of both languages is possible under the provision of “highly structured, person-centred, and frequently repeated input” (55). Children with a combined diagnosis of Down Syndrome and ASD exhibit language profiles similar to those of children with Down syndrome exclusively. However, these profiles are dependent on the exposure of the children to the respective languages (53). Studies also show that children with ASD can acquire a societal language in the same way as typically developing children (56). Some case studies even report positive effects of multilingualism on the social-communicative skills of children with ASD (57, 58). Despite the still limited research in many areas, studies on children with Down syndrome and ASD research suggest that children with ID can acquire multiple languages (37, 54). Another study on the influence of nonverbal IQ in the BIF range also shows little to no influence on language development in multilingual children (59). Nevertheless, parents of children with cognitive impairments are often advised to choose only one language (mainly the majority language) to avoid overwhelming the child (60, 61).

Since there are no tests to identify DLD specifically developed for this target group, adjustments are also proposed in this case. A comprehensive concept is provided by the “Approach for the Language Diagnosis of Multilingual Children with Cognitive Impairments” (47). The modified development-oriented approach consists of three steps: “Parent interview”, “Assessment of communicative and linguistic abilities in all languages”, and “Analysis of other language-relevant skills”. This approach is a proposal by the authors and aims to capture the child's language proficiency as accurately and precisely as possible in order to develop support measures based on it. In doing so, the individual cognitive abilities, disability-specific factors, and language acquisition circumstances should be taken into account (47).

In the lack of standardized tests for specific diagnoses, the role of SLPs becomes more important. SLPs therefore have a special responsibility to make a diagnosis using non-standardized procedures. This challenges their professional competence.

### 1.6 Self-perception of diagnostic competence

Self-perception of competence refers to an individual's perception of their own ability to perform tasks successfully. It reflects a person's subjective evaluation of their competence in a particular domain, rather than an objective assessment of their skills.

This concept lies at the intersection of actual competence—the observable ability to perform effectively with focus on the results of work tasks or job outputs (62)—and self-efficacy, which is the belief in one’s capacity to succeed in specific situations (63).

Since there is a positive connection between job competence and self-perceived competence, especially for job-specific skills (64, 65), assessing professionals’ perceived competence can provide insights into their actual competence. It can also reveal whether there is a general uncertainty among professionals in a particular area. This uncertainty could be addressed through targeted research, adjustments to training and study content, and continuing education (66).

Thomas et al. (67) investigated the experiences of SLPs in the assessment and diagnosis of DLD. They identified factors in assessment as barriers: individuality of impairments, choosing appropriate assessment tools, key indicators, and identifying non-language difficulties, which suggest limited self-perceived competence with regard to these factors.

Several surveys have been conducted in which SLPs were invited to share their diagnostic procedures for diagnosing DLD in multilingual children and to rate their perceived competence in this area (1, 66, 68, 69). These studies are comparable to the present investigation in which multilingual children also deviate from the monolingual “standard” language, and no specific testing procedures or normative data exist for them. Scharff Rethfeldt et al. (69) found that most clinicians felt confident in assessing multilingual children. The survey by Smith et al. (1) revealed little knowledge and/or use of standardized DLD tools, but relatively high self-perceived competence when working with multilingual children for two of the three countries. Lüke & Ritterfeld (66) and Marxen et al. (68) reveal significant uncertainty among SLPs in the diagnostic process and in selecting appropriate testing procedures for multilingual children. While Lüke & Ritterfeld (66) identified factors influencing perceived competence, namely their own multilingual socialization and independent professional development through specialist literature, Marxen et al. (68) did not identify influencing factors investigating professional experience, education, further training, further education through specialist literature or own multilingualism.

To the authors’ knowledge, there are no studies on the perceived competence of SLPs in the diagnosis of children with ID, neither monolingual nor multilingual.

### 1.7 Research goals

In summary, the state of research in the areas outlined above can be regarded as inconsistent and unsatisfactory. Furthermore, the diagnosis of DLD in children with ID can be described as challenging, as this patient group is characterized by a high degree of variability and with the obstacle that comprehensive tests and normative values for this target group are missing. In addition, there is disagreement among German-speaking countries regarding the terminology used for DLD in children with BIF (4). The present study aims to present the status quo of how practitioners currently deal with these challenges.

Three overarching questions were therefore examined on the basis of various sub questions in the survey.

1. What are the current practices of SLPs in assessing DLD with ID in German-speaking countries?
2. How does multilingualism change the practices of SLPs in the DLD assessment of children with ID?
3. How competent do SLPs feel in the DLD diagnosis of children with ID, and what influences this self-perceived competence?

The data are expected to provide direction to research, test development, education and trainings to enhance the approach to DLD diagnostics for children with ID, thereby providing practitioners with greater confidence in this field.

## 2 Methods

### 2.1 Design

Data were collected from practitioners who assess language skills in children with (suspected) ID using an internet-based cross-sectional survey. The survey conforms to the CROSS checklist for reporting surveys (70).

### 2.2 Survey development and structure

The survey was designed in three different steps by the authors of the study. First, German AWMF-S3 guideline on the treatment of DLD (32) and a widely used overview for diagnostic tools in Germany (Testzentrale.de) were used to design questions about the diagnostic procedures. Also, survey studies on DLD diagnostic procedures for multilingual children—even though these did not include questions about children with cognitive impairments (68, 69)—were employed to address additional questions concerning multilingualism and the assessment of competence. In the second step, the survey was piloted with a convenience sample of researchers and practitioners specializing in language diagnostics to assess its usability and technical functionality. Final revisions were made to enhance readability, including the addition of information boxes containing definitions cognitive impairments to ensure that everyone had the same understanding of the questions. The following information text was provided:

An IQ below 70 is generally considered indicative of an intellectual disability (ID) (WHO, 2022). An IQ between 70 and 85 is typically described as having “borderline intellectual functioning” or a “learning disability”. However, children with an IQ <85 also exhibit cognitive and language difficulties and are often excluded from the standardization of testing procedures (8, 10, 60). Therefore, these children are also included in this survey.

It is possible that there is no IQ test result available at the time of the language development assessment. Please also respond with regard to children who have cognitive impairments and a suspected “learning disability or ID”.

⇒ In the following questionnaire, “(suspected) ID” refers to these three groups: 1) IQ < 70; 2) IQ < 85; 3) Suspected 1) or 2).

For the purposes of this survey, this broader definition was used to reflect the clinical population encountered in language assessment, where an IQ result may not yet be available. Although the two IQ ranges are diagnostically distinct, they were therefore considered jointly in the questionnaire.

The survey was hosted online using LimeSurvey GmbH (71), version 6.14). It contained 38–42 questions divided into four sections: The first set of 18 questions asked participants about their current assessment practices, including how they evaluate DLD in children with an ID:

- At what point do practitioners apply DLD diagnostics on children with ID during treatment?
- What is their goal in applying the diagnostics?
- Do they use diagnostic tools that were developed for children with or without ID?
- Do they only include linguistic abilities in the diagnosis? Or do they also include non-linguistic abilities, and if so, which ones?
- Do they use normative values, and for what reason?
- Do they proceed differently with children with BIF than with children with ID? If so, how?

The second section contained nine questions about how their assessment practices change for multilingual children:

- Do practitioners use different diagnostic tools for multilingual children with ID than for monolingual children, and if so, why?
- Do they use normative values, and if so, which ones?
- In which language do they assess the children's verbal abilities?
- What do they think about the influence of multilingualism on language development in children with ID, and how did they come to this opinion?
- What recommendations do they give parents regarding the use of multiple languages?

The third section of the survey used nine items divided into two categories to assess participants’ personal sense of competence in this diagnostic field based on Marxen et al. (68):

- How competent do they feel in DLD diagnostics for children with ID? (e.g., how confident/insecure/challenged do they feel during the diagnostic procedure)
- How do practitioners obtain information about topics and methods in this field? (e.g., through literature or professional development courses)

The final section contained 14 questions to gather information about the participants’ demographics, education, clinical experience and the composition of their clinical caseload.

Question types varied, requiring responses in the form of multiple choice or open text. The questions regarding perception of competence utilize 5-point Likert scales to ensure comparability with the study by Marxen et al. (68), which they are based on. Definitions of key terms (see above) were provided at the beginning of each section to ensure consistent understanding. Completion of the survey took around 10 to 15 min in total. If they wished to modify their answers before submitting, respondents were able to navigate forward and backward between pages. No sensitive personal data, such as name, address or date of birth, was requested. Cookies and IP addresses were also not collected in order to maintain the anonymity of the responses.

### 2.3 Recruitment

Open invitations to complete the survey were sent out, containing the author's name, a link to the survey, and a brief description of the research purpose. Two distribution methods were used: 1) The invitation was sent to several mailing lists covering a broad network of practitioners working in the field of children's language therapy. These lists included associations of speech and language pathologists, researchers, and other practitioners: Gesellschaft für interdisziplinäre Spracherwerbsforschung und kindliche Sprachstörungen im deutschsprachigen Raum e.V. [Society for Interdisciplinary Research on Language Acquisition and Childhood Language Disorders in German-Speaking Countries] (GISKID), Deutscher Bundesverband für Logopädie e.V. [German Federal Association for Speech Therapy] (DBL), and Deutscher Bundesverband für akademische Sprachtherapie und Logopädie [German Federal Association for Academic Speech and Language Therapy and Logopedics] (DBS) as well as alliances of early intervention centers, asking them to share it with their members. 2) The invitation was distributed on social media. The latter method involved sending a private message to SLP-related Instagram accounts, requesting that they post the invitation in their feed or story. It was also posted in several professional Facebook groups. Before starting the survey, participants had the opportunity to read about the purpose of the research, anonymity, data storage arrangements, and the suggested length of time required. Participation was voluntary and no incentives were offered for completion; participants were also free to withdraw at any time. A total of 220 participants opened the survey and provided informed consent, of whom 102 completed the survey up to the final page. Thus, 118 participants did not complete the survey. Most drop-outs occurred after participants had read the definitions of ID and completed the first set of questions concerning the diagnostic procedure. One possible explanation is that the recruitment email lists included practitioners working with children in general, rather than exclusively practitioners working with the intended client population. Consequently, some participants may have determined at this stage that the survey was not relevant to their professional practice. As the specific reasons for individual drop-outs were not assessed, this explanation should be considered tentative. The final sample therefore comprised the 102 participants who completed the survey (*N* = 102).

### 2.4 Sample

#### 2.4.1 Personal characteristics

Participants were predominantly aged 31–40 years (33.3%), followed closely by those aged 41–50 years (31.4%). The 20–30-year age group accounted for 18.6% of the sample, while 16.7% of participants were aged 51 years or older. Most participants identified as female (94.1%), while 5.9% identified as male. No participants identified as non-binary/diverse. One participant did not disclose their gender.

#### 2.4.2 Professional characteristics

Participants represented a broad range of professional backgrounds, educational qualifications, institutional settings, and levels of professional experience. Detailed participant characteristics are presented in Table 1.

**Table 1:** Professional characteristics of participants (*N* = 102).

Characteristic	Category	*n* (%)
Professional background	Speech-language therapists (Logopäd:innen)	61 (59.8)
	Clinical linguists	10 (9.8)
	Special needs educators of speech and language (Sprachheilpädagog:innen)	15 (14.7)
	Teachers	5 (4.9)
	Psychologists/neuropsychologists	4 (3.9)
	Other professions	20 (19.6)
Highest educational qualification	Master's degree	32 (31.4)
	Vocational training with state examination	27 (26.5)
	Bachelor's degree	18 (17.6)
	Doctorate (PhD/Promotion)	6 (5.9)
	Other qualification including state examination	19 (18.6)
Institutional setting	Early intervention services	46 (45.1)
	School settings	41 (40.2)
	Speech and language therapy practices	27 (26.5)
	Early childhood education/daycare	19 (18.6)
	Social pediatric centers	15 (14.7)
	Universities/advisory services/other	6 (5.9)
	Phoniatrics/pediatric audiology	3 (2.9)
	Autism therapy centers	2 (2.0)
	Hospital/rehabilitation clinic	1 (1.0)
Professional experience in speech and language diagnostics	0–5 years	21 (20.6)
	6–10 years	20 (19.6)
	11–15 years	24 (23.5)
	16–20 years	17 (16.7)
	>20 years	20 (19.6)
Professional experience with children with (suspected) ID	0–5 years	27 (26.5)
	6–10 years	26 (25.5)
	11–15 years	19 (18.6)
	16–20 years	15 (14.7)
	>20 years	15 (14.7)

Multiple responses were possible for all categories. “Other professions” included, for example, special education teachers, rehabilitation educators, mototherapists, social educators, hearing and language specialists, and researchers/scientists. “Other qualification” included diplomas, Magister degrees, and state examinations. The variety of highest educational backgrounds also reflects the different professional roles in the field of child language, where academic degrees coexist with traditional vocational training in Germany.

#### 2.4.3 Continuing professional development

Practitioners reported on the extent to which they keep up to date through professional literature or attend trainings. About 70% of respondents indicated that they consult professional literature on the assessment of children with DLD and (suspected) ID at least partially, with 13.9% reporting full engagement. Similarly, 66% attend trainings on language development in children with (suspected) ID at least partially, with 15.7% participating fully. Trainings on related topics were particularly common: 65.3% attended such trainings at least partially, and 31.6% reported full participation. Overall, trainings on related topics appear to be the most frequently utilized form of professional development among the surveyed practitioners.

#### 2.4.4 Geographical distribution

Participants were recruited across the DACH region, including Germany, Austria, and Switzerland. The majority of respondents were working in Germany (97.1%), while smaller proportions reported working in Switzerland (2.0%) or Austria (1.0%). Thus, all three DACH countries were represented in the sample. The strong predominance of participants from Germany reflects differences in professional structures and recruitment opportunities across the region. Many child language–related organizations and professional networks in Switzerland and Austria are closely aligned with German organizations, resulting in fewer independent mailing lists and a more limited reach for direct recruitment in these countries. Consequently, recruitment efforts yielded a substantially larger number of participants from Germany.

Within Germany, participants were distributed across a wide range of federal states. The largest shares were from North Rhine-Westphalia (39.2%) and Bavaria (23.5%). Further participants were recruited from Hesse (6.9%), Lower Saxony (6.9%), Baden-Württemberg (4.9%), and Saxony (4.9%). Smaller numbers of respondents were from Bremen (2.0%), Mecklenburg–Western Pomerania (2.0%), and Schleswig-Holstein (2.0%), as well as from Brandenburg (1.0%), Hamburg (1.0%), Rhineland-Palatinate (1.0%), Saxony-Anhalt (1.0%), and Thuringia (1.0%). In addition, 2.9% of participants reported working in other federal states. The participant working in Austria was located in Vienna, while the two participants working in Switzerland were based in the cantons of Basel and Aargau, respectively.

#### 2.4.5 Caseload demographics

Participants reported working with children across a broad range of age groups. The majority of professionals indicated that their caseload included children aged 4–6 years (96.1%), followed by children aged 0–3 years (68.6%). Slightly more than half of the participants reported working with children aged 7–11 years (54.9%). In contrast, fewer participants indicated working with older age groups: 29.4% reported including adolescents aged 12–16 years in their caseload, and only 13.7% worked with individuals aged 17 years and older.

Participants reported assessing the language development of children with a broad spectrum of comorbidities and underlying conditions, reflecting a highly heterogeneous caseload. Table 2 provides a detailed overview of the reported diagnoses of the assessed children.

**Table 2:** Comorbidities and underlying conditions of the assessed children.

Diagnosis	*n* (%)
Global developmental delay	94 (92.2)
Preterm-born	87 (85.3)
Social-emotional support needs	88 (86.3)
Diagnosed autism spectrum disorder	90 (88.2)
Suspected autism spectrum disorder	91 (89.2)
Rett syndrome	36 (35.3)
Down syndrome	76 (74.5)
Other genetic syndromes	75 (73.5)
Fetal alcohol spectrum disorder	75 (73.5)
ADHD	94 (92.2)
Dyslexia	44 (43.1)
Dyscalculia	28 (27.5)
Hearing impairment	61 (59.8)
Epilepsy	62 (60.8)
ID of unclear etiology	89 (87.3)
Other	3 (2.9)

Participants were invited to estimate the proportion of children in their respective assessments for language development who have or are suspected of having an ID. The largest group of respondents indicated that between 26% and 50% of their caseload fell into this category (33.7%), followed closely by those reporting 1%–25% (29.7%). Smaller proportions reported that 51%–75% (18.8%) or 76%–99% (11.9%) of the children they assess had an ID or suspected ID, while only 5.9% of respondents indicated that all children (100%) in their caseload were affected. When asked how many of these children had an IQ test result available, only a small proportion of participants reported that an IQ test was available for all children (9.8%), while about one quarter indicated that it was available for most children (24.5%). A larger group reported that an IQ test was available for some children (41.2%). In contrast, 17.6% of participants indicated that an IQ test was usually not available, and 6.9% selected “Other”, providing alternative descriptions.

For 24 participants (24%), the proportion of children with (suspected) ID who grew up multilingual was between 1% and 25%. A total of 31 participants (31%) reported that 26%–50% of the children were multilingual, and 31 participants (31%) indicated that 51%–75% of the children grew up multilingual. Finally, 14 participants (14%) stated that 76%–99% of the children in this group were multilingual. Responses to this question were available for 100 of the 102 participants.

## 2.5 Data analysis

Descriptive statistics were used to analyze the data from the multiple-choice and Likert response questions. Analyses were performed on all responses received, with no values substituted for missing data. A multiple linear regression was conducted to examine predictors of the competency score, including age, work institution, experience score and a knowledge acquisition score.

The experience score was developed to capture different dimensions of the participants’ practical experience relevant to the assessment of children with ID. It comprised professional experience in the field of DLD diagnostics (in years), experience in working with children with ID (in years), and the proportion of children with ID in the respective institution in which they worked (in percentages). These three indicators were considered complementary aspects of practical experience, reflecting both the duration of professional experience and the frequency of contact with the target population. Each item was assigned a score from 1 to 5, with 1 point representing a low level of experience or a small proportion of children with ID and 5 points representing a high level of experience or a large proportion. The individual components were equally weighted because no empirical basis was available for assigning different weights to the three dimensions. The points were then aggregated to yield a composite Experience Score, with higher scores indicating greater and broader practical experience with DLD diagnostics and children with ID. As this composite measure was developed specifically for the present study, its construction should be considered exploratory.

The Knowledge Acquisition Score comprised three items addressing whether practitioners informed themselves about DLD diagnostics in children with ID through specialist literature, attended training courses on DLD diagnostics in children with ID, or attended training courses on related topics. The items and the 5-point Likert scale were based on Marxen et al. (68). In the present study, these items were combined to form a composite Knowledge Acquisition Score, with higher scores indicating more frequent engagement in professional knowledge acquisition. The items were equally weighted and summed to obtain a cumulative score. As Marxen et al. (68) did not combine these items into a cumulative score, the composite Knowledge Acquisition Score was developed specifically for the present study and should therefore also be considered exploratory.

The regression model was estimated via ordinary least squares with all predictors entered simultaneously. Assumptions were checked by inspecting residuals and variance inflation factors (VIF < 2). Model fit was evaluated using R^2^ and adjusted R^2^. The initial model included many predictors relative to the sample size, increasing complexity and risk of overfitting. Conceptually related variables were therefore combined in a reduced model (e.g., institutional settings grouped into therapy center, school, and clinic), resulting in fewer parameters (df = 13 vs. 21) and lower information criteria (AIC = 568.55, BIC = 602.67 vs. AIC = 575.13, BIC = 630.26). While the original model explained slightly more variance, the adjusted R^2^ values were comparable, supporting the use of the more parsimonious reduced model.

All analyses were conducted in R (v4.3.3; (72) using *emmeans* (73), *car* (74).

The open-text responses were analyzed using qualitative content analysis following Mayring's summarizing approach (75). All responses were read repeatedly, and relevant meaning units were identified and paraphrased. Similar paraphrases were generalized, reduced, and merged. Categories were developed inductively from the data without a predefined coding scheme. Semantically related content was grouped into main categories, which were continuously reviewed and refined. One response could be assigned to more than one category. The frequency of mentions per category was recorded to indicate their relative relevance within the data.

## 3 Results

### 3.1 Diagnostic procedure of DLD in children with (suspected) ID

To obtain a comprehensive picture of how practitioners proceed with DLD diagnostics when a child has a (suspected) ID questions about the timing of the assessment, the intended goals, whether the participants apply the same criteria as for DLD, which language-specific and additional diagnostic procedures they use, and also how they deal with standard values were asked.

#### 3.1.1 Timing and goals of language assessment

For the following questions participants had the opportunity to choose responses from an array of multiple choice alternatives with multiple answers allowed. Analysis of the answers about the timing of the assessment procedure indicated that practitioners primarily apply these measures at the beginning and during the course of care. The majority of all respondents (*N* = 102) reported using screening or diagnostic procedures for initial assessment (86.3%, *n* *=* 88), followed by progress monitoring (78.4%, *n* = 80). Use for final assessment was less common (49.0%, *n* = 50). Screening and diagnostic procedures were used for reports for physicians by 45.1% (*n* = 46) of practitioners, for schools by 29.4% (*n* = 30), and for daycare/kindergarten by 18.6% (*n* = 19). Only a small proportion of respondents indicated that they do not use screening or diagnostic instruments at all (2.9%, *n* = 3).

Regarding the goals of language assessment, practitioners most frequently reported that their primary objective is to identify specific areas of impairment within language (90.2%, *n* = 92). A substantial proportion also indicated that assessment is used for treatment planning (73.5%, *n* = 75). Further commonly endorsed goals included identifying potential accompanying or influencing factors on language development (65.7%, *n* = 67) and the evaluation of therapy outcomes (64.7%, *n* = 66). In contrast, a good half of respondents reported that a core goal of assessment is disorder classification, i.e., determining whether a developmental language disorder is present or not (56.9%, *n* = 58).

#### 3.1.2 Usage of tools designed for children with or without ID

The analysis of the question “When assessing the language development of a child with (suspected) ID, I use … tools that have been developed for children with or without ID” show a clear overall pattern: when assessing the language development of children with (suspected) ID, practitioners predominantly use instruments that were originally developed for children without ID, while tools specifically developed for children with ID are used only rarely. Table 3 shows that this pattern is consistent across anamnesis forms, standardized parent questionnaires, and diagnostic/screening procedures, with the strongest contrast observed for standardized diagnostic tests. As previously outlined, there is a paucity of diagnostic materials for children with ID in Germany. Participants could thus provide further details in a free-text field about which materials they were referring to. Four individuals reported adapting tests based on Aktas (38); one person also used the Test of Aided-Communication Symbol Performance (TASP (76); the others did not provide any further details.

**Table 3:** Instrument types used for assessing language development in children with (suspected) ID (*N* = 102).

Instrument type	Developed for children without ID—Yes *n* (%)	Developed for children with ID—Yes *n* (%)
Anamnesis forms	80 (78.4%)	13 (12.7%)
Standardized parent questionnaires	78 (76.5%)	11 (10.8%)
Diagnostic tests/screenings	100 (98.0%)	9 (8.8%)

#### 3.1.3 Inclusion of nonlinguistic skills in the diagnostic process

The vast majority of participants (94.1%) disagreed with the statement that only linguistic competencies should be considered in the diagnostic process, while 5.9% reported an exclusively language-focused assessment. Correspondingly, 95.1% of respondents indicated that they also consider non-linguistic abilities when evaluating language development, whereas 2.9% reported not to include such abilities in their assessment process.

Participants were invited to elaborate on the nonverbal skills they incorporate into the diagnostic process through open-text responses. The analysis shows that respondents frequently consider non-linguistic abilities when assessing the language development of children with (suspected) ID (see Table 4). The most frequently mentioned category is attention and concentration (*n* = 68), indicating that attentional capacities are viewed as a key prerequisite for valid language assessment. Memory and information processing abilities, particularly short-term memory, memory span, and working memory, are often mentioned (*n* = 54), highlighting their relevance for both language comprehension and task performance during assessment situations. Closely related, executive functions and cognitive control processes such as planning, impulse control, and task understanding are reported by a substantial number of respondents (*n* = 31). General cognitive abilities and overall developmental level, including references to intelligence and global development, are mentioned in many responses (*n* = 30). In addition, perceptual and sensory prerequisites, especially auditory perception and hearing status, are considered relevant by a notable proportion of participants (*n* = 26). Motor abilities, including gross motor, fine motor, and orofacial motor skills, are mentioned somewhat less frequently but still consistently across responses (*n* = 24). Social-emotional and motivational factors such as motivation, interests, emotional development, and resilience are also taken into account by many respondents (*n* = 27). Furthermore, interactional and pragmatic aspects of communication, including turn-taking and social interaction, are frequently referenced (*n* = 33). Preverbal abilities and language precursors such as eye contact, joint attention, gestures, and imitation are highlighted by a large number of participants (*n* = 36), underlining their importance for early language development. Play behavior and play development are mentioned as diagnostic indicators by several respondents (*n* = 22). Finally, contextual and environmental factors, including setting, interaction with caregivers, and language input, are explicitly considered in a smaller but relevant number of responses (*n* = 15).

**Table 4:** Additional competencies which practitioners include into language assessments of children with (suspected) ID.

Main category	Description	Example anchor quotes	Mentions (*n*)
Attention & concentration	Ability to maintain, focus, and regulate attention during tasks	“Attention, concentration”, “attentional focus, endurance”	68
Memory & information processing	Short-term memory, memory span, working memory, processing speed	“Memory span, working memory”, “short-term memory in different modalities”	54
Executive functions & cognitive control	Planning, impulse control, task understanding, cognitive flexibility	“Executive functions”, “action planning”, “impulsivity in problem solving”	31
General cognitive abilities & development	Overall cognitive level, intelligence, developmental status	“Cognitive development”, “IQ”, “overall development”	30
Perception & sensory prerequisites	Auditory, visual, and multisensory perception	“Auditory perception”, “hearing status”	26
Motor abilities	Gross motor, fine motor, oral motor, sensorimotor skills	“Gross and fine motor skills”, “orofacial motor skills”	24
Social-emotional & motivational factors	Emotional development, motivation, interests, resilience	“Motivation”, “interests of the child”, “emotional development”	27
Interaction, pragmatics & social communication	Social use of communication, pragmatics, turn-taking	“Interaction”, “pragmatics”, “turn-taking”	33
Preverbal abilities & language precursors	Eye contact, joint attention, gestures, imitation, symbolic understanding	“Eye contact and triangulation”, “language precursors”	36
Play behavior & play development	Play as an indicator of cognitive and linguistic development	“Play behavior”, “play development”, “symbolic play”	22
Contextual & environmental factors	Setting, environment, caregiver interaction, language input	“Setting”, “interaction with caregivers”	15

#### 3.1.4 Usage of normative values

Most respondents reported that they use normative values when interpreting test results for children with (suspected) ID. Specifically, 61.8% of practitioners (*n* = 63) indicated that they rely on normative values in their evaluations, whereas 38.2% (*n* = 39) reported that they do not use norms when assessing this population.

Practitioners who reported using normative values (*n* = 63) indicated multiple purposes for their use, which they chose from multiple choice alternatives. The majority of those participants stated that they apply norms to obtain a general orientation regarding the child's language abilities (76.2%, 48/63) and to compare children's language performance with age-appropriate normative values (77.8%, 49/63). More than half of the respondents (57.1%, 36/63) reported that they use normative values to compare language performance with the child's mental or developmental age rather than her or his chronological age. In contrast, fewer respondents (42.8%, 27/63) indicated that they use normative values primarily for the purpose of diagnosing a language disorder.

Among practitioners who reported not using normative values (*n* = 39), the most frequently endorsed reason was that children with ID cannot be meaningfully compared to age-based norms developed for children with typical development (79.5%, 31/39). In addition, 38.5% (15/39) of respondents agreed that age norms for children with ID are generally misleading. Nearly half of the respondents (46.2%, 18/39) indicated that, given that children with ID often present with concomitant speech and language difficulties, formal disorder-based diagnostics (e.g., presence vs. absence of a speech or language disorder) are considered less relevant than qualitative assessment and the identification of individual support needs. Only one individual (2.6%, 1/39) expressed a lack of familiarity with normative values.

#### 3.1.5 Approaches for different IQ-groups

The analysis of free-form responses regarding diagnostic approaches for children with different levels of cognitive impairment shows a heterogeneous practice among professionals (see examples and frequencies of the answers in Table 5). Several respondents adjust their assessment depending on the child's IQ or type of cognitive impairment, particularly for children with ID (IQ < 70), where informal, observation-based approaches and shorter testing sessions are preferred.

**Table 5:** Adjustments of diagnostic procedure depending on IQ-groups.

Main category	Description	Example anchor quotes	Mentions *(n)*
Adjustment based on IQ/impairment level	Adjustments of diagnostic procedures depending on child's IQ level, developmental age, or severity of impairment (adjustments stronger for children with IQ < 70 than for children with IQ 70–85)	“Yes, test procedures are selected according to developmental age or adapted downward to determine language age.”/“The lower the IQ, the more I would rely on spontaneous language analysis instead of standardized tests.”/“There are clear differences in learning abilities between a child with an IQ of 63 and one with an IQ of 82.”	48
Test selection & adaptation	Modifying tests, selecting easier or developmentally younger test versions, reducing task difficulty, shortening sessions, changing the setting, or using more informal procedures	“I would adapt the testing situation and use fewer standardized procedures, relying more on spontaneous speech and observation.”/“Use of the SETK-2 even with older children if higher-level tests would be overwhelming.”/“The child should feel capable of completing the tasks—start at a low level and use good stopping criteria.”	42
Qualitative vs. quantitative approach	Preference for developmental profiles, observation, spontaneous speech analysis, or other qualitative methods over purely standardized scores	“A qualitative evaluation makes more sense; I would rather calculate developmental age than a T-score.”/“I focus on everyday relevance and communication in general.”/“If an IQ diagnosis exists, I would evaluate tests more qualitatively.”	26
Contextual/individual factors	Child-specific or environmental influences on diagnostic approach (e.g., attention, motivation, endurance, emotional state, participation)	“It depends on the child and what they need.”/“I orient myself toward the child's abilities and the diagnostic question.”/“The approach always depends on the child's performance and cooperation.”	34
Cooperation & external support	Involving colleagues or obtaining second opinions, wishing for better diagnostic tools and external support structures	“Involve a colleague from a school for intellectual development.”/“Own IQ testing and, if in doubt, a second opinion from a special education colleague.”/“If there were specialized tests available, I would use them.”	10
No or minimal adjustment	Standardized procedures applied regardless of cognitive profile	“Currently not.”/“No, I always orient myself to the child's current developmental level.”/“No, because in all cases I consider qualitative evaluation more meaningful.”	24

For children with IQ between 70 and 85 (“learning disability” or BIF) or suspected cognitive difficulties, many practitioners still apply standardized tests but adapt the difficulty, material, or duration to the child's developmental level, attention, and motivation. A substantial number of respondents emphasize the use of qualitative or developmental approaches, such as calculating developmental age, spontaneous speech analyses, and observational methods, rather than relying solely on normative test scores. Contextual and individual factors, including attention, motivation, social-emotional engagement, and the testing environment, are also reported to influence the choice and adaptation of diagnostic procedures. Some respondents involve external professionals, for example colleagues from special schools, for children with more severe impairments.

At the same time, a notable group reports little or no differentiation in their diagnostic approach, relying on standard procedures and adapting only minimally to the child’s specific cognitive profile.

### 3.2 Multilingualism in the context of DLD and ID

The next section of the questionnaire asked how multilingualism should be dealt with in cases of (suspected) ID.

When asked whether they use different diagnostic procedures for children with (suspected) ID who are raised bilingually versus monolingually, the majority of respondents (84.3%, *n* = 86) reported that they do not, while 15.7% (*n* = 16) indicated that they do use different procedures. Participants were invited to select reasons from a set of multiple-choice answers: Among those who use the same procedures, 51.2% (44/86) stated that they interpret the results qualitatively, 7.0% (6/86) considered the procedures equally suitable for both groups, 7.0% (6/86) did not see monolingual versus bilingual status as relevant for test selection, 15.1% (13/86) highlighted the good control of standardized materials (whereby “materials” were not specified in more detail here), 77.9% (67/86) indicated that no alternative procedures are available, and 52.3% (45/86) described their approach as a compromise solution.

For the following questions participants (*N* = 102) were presented with multiple-response items and could select more than one answer where applicable. Regarding the use of normative data in the assessment of bilingual children with (suspected) ID, 42.2% of respondents reported using norms developed for monolingual children, 29.4% use norms adjusted for the duration of exposure to the societal language, and 39.2% indicated that they do not use any normative data.

In terms of the language used to assess children's linguistic abilities, 44.6% of respondents always use German, 9.9% use both languages, and 45.5% use German and the child's first language when possible. When practitioners do not speak the child's first language, 46.4% seek assistance from another professional who speaks the language, 67.9% use translated parent questionnaires or checklists, and 33.9% work with an interpreter during assessment. Among respondents who do not include the child's first language in the evaluation, 60.4% reported that they cannot professionally assess the language themselves, and 33.0% indicated that they are not aware of procedures available for testing the child's first language.

In relation to the perceived impact of multilingualism on the verbal development of children with (suspected) ID, participants could select multiple responses. Accordingly, 28.4% of respondents considered the effect to be positive, 47.1% considered it to be negative, and 45.1% expected no impact to be observed. When explaining their responses, 75.5% of respondents referred to their own clinical experience, 30.4% cited findings from the literature, 29.4% referenced knowledge acquired during their studies or training, and 26.5% drew on information from continuing education or professional development courses.

Regarding recommendations given to parents of bilingual children with (suspected) ID about the use of the languages, nearly 58% of respondents endorsed the recommendation that parents should speak only the family language at home. In contrast, only 5% supported recommending that parents should speak the societal language as often as possible, and less than 1% indicated that no recommendation should be provided.

### 3.3 Self-perception of competence

Practitioners were asked to rate their self-perceived competence in the assessment of children with (suspected) ID on a 5-point Likert scale. The first five items addressed uncertainty about which diagnostic procedures to use, feelings of being overwhelmed during assessment, confidence in diagnosing and deriving interventions for children with DLD and ID, ease of capturing specific conditions in case history, and overall confidence in conducting assessments. Respondents indicated moderate confidence overall, with higher agreement for feeling confident in specific aspects of assessment and lower agreement for feelings of uncertainty or being overwhelmed (see Table 6 for details).

**Table 6:** Self-perceived competence in the assessment of children with (suspected) ID.

Item	Not at all (%)	Rather not (%)	Partially (%)	Rather yes (%)	Fully (%)	Total
I am uncertain about which diagnostic procedures to use	12.7	23.5	37.3	13.7	12.7	102
I feel overwhelmed during assessment	25.5	39.2	18.6	8.8	7.8	102
I feel confident in diagnosing and deriving interventions for a language development disorder	5.0	12.9	18.8	48.5	14.8	101
It is easy for me to capture the specific conditions of children in the case history	3.9	19.6	28.4	34.3	13.7	102
I feel confident in conducting assessments	5.9	16.7	24.5	40.2	12.7	102

#### 3.3.1 Influencing factors on self-perception of competence

A multiple linear regression was conducted to examine predictors of the competency score, including experience, knowledge, demographic variables, and institutional settings. The reduced model included conceptually grouped institutional variables (specialized therapy centers, school, and clinic) alongside experience score, knowledge acquisition score and age. The overall regression model was statistically significant and explained 23% of the variance in competency scores [*R*^2^ = 0.23, Adjusted *R*^2^ = 0.14, *F* (11, 90) = 2.50, *p* = .009].

Significant positive predictors included experience (*β* = 0.46, *p* = .009) and affiliation with a therapy center (*β* = 2.29, *p* = .037), indicating that higher experience and working in a therapy center were associated with higher competency scores. Other predictors, including knowledge, age groups, school and clinic settings, and other institutional affiliations, were not statistically significant in this model (see Table 7).

**Table 7:** Regression results for competency score.

Predictor	Estimate (*β*)	Std. Error	*t*	*p*
Intercept	9.87	2.25	4.39	<.001 ***
Experience Score	0.46	0.17	2.67	.009 **
Knowledge Acquistion Score	0.17	0.16	1.04	.301
Therapy Center	2.29	1.08	2.12	.037 *
Practice	0.64	1.05	0.61	.545
School	0.44	1.01	0.44	.663
Clinic	−1.37	1.93	−0.71	.479
Kindergarten	−0.58	1.00	−0.58	.561
Other Institution (Consulting, University, Psychology Practice)	1.70	1.81	0.94	.350
Age 31–40	0.17	1.17	0.15	.885
Age 41–50	0.09	1.37	0.07	.946
Age >50	1.33	1.66	0.80	.427

## 4 Discussion

### 4.1 Diagnostic procedure of DLD in children with (suspected) ID

In the present study, the goal was to provide an overview of the current clinical practice in language assessment for children with (suspected) ID. Therefore, an online survey was conducted with *N* = 102 participants and was evaluated with respect practices among SLPs in diagnosing children with (suspected) ID, their appraisals and adjustments concerning multilingualism and ID, their personal sense of competence and influencing factors on it.

Results regarding the timing and goals of language assessment show that practitioners most frequently use diagnostic instruments at the beginning of intervention and for progress monitoring, whereas final evaluations are emphasized less often. Similarly, respondents most often described the identification of specific language difficulties and treatment planning as central goals of assessment, while fewer practitioners considered categorical disorder classification to be a primary objective. Taken together, these findings suggest that practitioners conceptualize language assessment in children with (suspected) ID primarily as a holistic, process-oriented, and individualized procedure rather than as a purely classificatory diagnostic process which is largely in line with the guidelines on treatment of DLD in Germany (32).

With regard to the reported use of diagnostic instruments, practitioners relied mostly on tools originally developed for children without ID, whereas instruments specifically designed for children with ID were used only rarely. This can be explained by the lack of valid assessment materials for this population in the German-speaking context (see chapter 1.4). This is emphasized by the fact that even among those who cited tools specifically designed for children with ID, only five explanations were given and four of these named test adaptations based on Aktas (38), as also described above.

At the same time, practitioners reported adopting a broad and multidimensional understanding of language assessment. Nearly all respondents indicated that they consider non-linguistic abilities in addition to linguistic competencies when evaluating language development of children with (suspected) ID. Frequently mentioned domains included attention, memory, executive functions, general cognitive abilities, preverbal skills, pragmatic competencies (as a non-structural linguistic domain), and play behavior. This is in line with the ID-definitions and description of symptoms provided by AAIDD (6) and WHO (5). It indicates that practitioners adopt a holistic and developmental perspective on communication rather than focusing exclusively on formal linguistic structures. While multidimensional approaches may support individualized intervention planning, the broad range of additional competencies assessed also points to significant heterogeneity in diagnostic practice.

In the reported handling of normative values most practitioners indicated that they use overall norms when interpreting assessment results. These were primarily employed for descriptive orientation and contextualization rather than for formal diagnosis. Many clinicians reported comparing language performance to developmental or mental age instead of chronological age, which is a method described in literature for dealing with these values in children with ID (37, 45, 46). At the same time, a substantial proportion of respondents questioned the clinical meaningfulness of comparing children with ID to age-based norms derived from typically developing populations and emphasized the greater relevance of qualitative assessment and individualized intervention planning. This also reflects an awareness of the absence of appropriate norms and tools.

### 4.2 Approaches for IQ < 70 vs. IQ between 70 and 85

The reported adaptations of diagnostic procedures across different IQ groups further reinforce the impression of a highly individualized clinical practice. Many practitioners described adjusting their assessment strategies according to the severity of cognitive impairment, with particularly strong adaptations for children with ID (IQ < 70). In these cases, clinicians often reported relying less on standardized testing and more on qualitative, observational, and developmentally oriented approaches, including spontaneous speech analysis, developmental profiling, and shortened or simplified testing procedures. In spontaneous speech analysis, however, children are also evaluated based on a target language standard. This involves comparing the observed utterances with those produced in typical language development in order to determine the child**’**s current stage of development at the time that the spontaneous speech sample was recorded (77). For children with an IQ between 70 and 85, standardized procedures were still frequently applied, although often in adapted form. Respondents described selecting test versions for younger children, modifying task difficulty, or adjusting session length and testing conditions according to the child**’**s attentional and motivational capacities, which is in line with Neitzel & Dittmann (37). Such adaptations indicate that even though clinicians are aware of the limitations of existing tools in this populations they try to balance standardization and attempt to maintain task accessibility.

Although the present survey included children with both ID (IQ < 70) and cognitive functioning in the IQ range of 70–85, the findings indicate that practitioners do not necessarily regard these groups as equivalent. The reported adaptations of diagnostic procedures according to cognitive impairment suggest that the severity of impairment influences clinical decision-making.

The finding that numerous SLPs differentiate between children with an IQ < 70 and those with an IQ between 70 and 85 when selecting diagnostic procedures reflects the disagreement among practitioners which is also shown in the German terminology study (4). In that DELPHI consensus study, experts were asked to clarify the definition criteria of language disorders in childhood and resolving conceptual ambiguities of terminology. The experts have reached a consensus on many points. Only on a few points it was not possible to reach a consensus, e.g., experts disagreed on whether the IQ range between 70 and 85 should be classified as “DLD” or “DLD associated with X”.

The SLPs’ statements in the present study suggest a similar perspective: although they regarded this IQ range as representing a milder impairment than IQ < 70, they still considered it sufficiently severe to preclude a pure DLD diagnosis (“DLD without X”) and instead favored the use of adaption. At the same time, a substantial proportion of respondents reported little or no differentiation in their assessment procedures across IQ groups. This limited differentiation may reflect the tendency in German-speaking surroundings to conceptualize BIF as closely related to, or even part of, ID, which may stem from the longstanding tradition of pathologizing the IQ range between 70 and 85 (8–10).

The involvement of external specialists by some respondents additionally suggests that practitioners may experience uncertainty when assessing children with ID and may seek interdisciplinary support to compensate for limited diagnostic resources or expertise.

### 4.3 Multilingualism in the context of DLD and ID

Although most respondents reported using the same procedures for bilingual and monolingual children with (suspected) ID, many described their approach as a “compromise” solution, indicating awareness that existing standardized instruments may not adequately capture multilingual language development in this population (47, 50). The most frequently reported reason for not adapting procedures was the absence of suitable tools.

The variation in the use of normative values and language choice during assessment further reflects often limited language competence and the lack of appropriate standardized materials (47).

The differences in expectations regarding the impact of multilingualism on language development suggest considerable uncertainty among practitioners about how multilingual exposure interacts with ID. Notably, respondents relied predominantly on their personal clinical experience rather than research evidence to explain their views, irrespective of whether they evaluated the influence of multilingualism in the language development of children with ID positively or negatively. Nevertheless, the recommendations provided to families showed a comparatively consistent pattern, with most respondents advising parents to continue using the family language at home. This finding is broadly consistent with current research on multilingualism emphasizing the importance of rich and emotionally meaningful language input in the home language (53, 55, 56). At the same time, the small proportion of practitioners recommending increased use of only one language suggests that uncertainties and potentially outdated assumptions regarding multilingualism and developmental disabilities may still persist in some areas of clinical practice as also described in Dott & Licandro (60) and Hernandez et al. (61).

### 4.4 Self-perception of competence

The findings on self-perceived competence indicate a generally moderate level of confidence among practitioners when assessing children with (suspected) ID. While respondents largely reported feeling confident in conducting assessments and deriving implications for intervention, a non-negligible proportion also indicated uncertainty regarding the selection of appropriate diagnostic procedures and variability in perceived ease of handling those cases. Similar observations have been made by SLPs during the diagnostic process with multilingual children (without ID) (1, 66, 68, 69). This pattern suggests that practitioners experience assessment in this field as demanding but not overwhelmingly difficult, which aligns with the previously described heterogeneity and lack of standardized procedures.

The regression results further highlight that self-perceived competence is primarily associated with professional experience. Practitioners with greater experience reported significantly higher self-perceived competence. This is not in line with Marxen et al. (68) who investigated competence in diagnosing multilingual children without ID: they did not identify professional experience as an influencing factor. In contrast, knowledge-related measures and demographic variables such as age did not contribute significantly. Lüke & Ritterfeld (66) instead found an influence of expansion of knowledge through specialist literature—but again regarding diagnosing multilingual children without ID. In addition, the results of the present study show that working in a specialized therapy center was positively associated with higher competence ratings, suggesting that institutional context may provide structured opportunities for skill development and case exposure. Overall, these findings indicate that competence in this diagnostic area appears to be largely practice-based and shaped by experiential learning rather than formal training or demographic characteristics.

However, these findings should be interpreted in light of the relatively limited explanatory power of the regression model. Although the model explained 23% of the variance in self-perceived competence, the adjusted R^2^ was.14, indicating that a substantial proportion of the variability in competence remained unexplained after accounting for the number of predictors included. Thus, professional experience and affiliation with a specialized therapy center may contribute to differences in self-perceived competence, but they should not be regarded as comprehensive determinants. Other factors not assessed in the present study may also contribute to practitioners’ perceived competence. Overall, the findings provide evidence for an association between experiential and institutional factors and self-perceived competence, while suggesting that competence is likely shaped by a broader range of factors than those captured by the present regression model.

### 4.5 Limitations

The composition of the sample can be evaluated critically. While the sample is relatively large, indicative of the relevance of the topic in SLP clinical practice, it is geographically unevenly distributed, with only very few single participants from Austria and Switzerland. In order to provide a more representative picture of the practices in these countries, a larger sample would need to be collected from these two countries. The heterogeneity of job titles and degrees also reflects the variability within the German system. Larger sample sizes for each occupational group would therefore have been desirable. Despite recruitment invitations addressed to any clinician diagnosing DLD in children with (suspected) ID, responses were mostly from experienced clinicians with advanced degrees. We may conclude that the respondent sample was biased toward practitioners with a high level of expertise. This may be reflected in the proportion of academic degrees held and years of professional experience. According to the data, 56% of the participants hold an academic degree rather than a state-certified qualification, and almost 60% have over 11 years’ experience. This sampling bias may have contributed to comparatively high self-perceived competence ratings and therefore limits the generalizability of these findings to the broader SLP population.

The relatively high number of drop-outs should also be considered when interpreting the findings. Of the 220 participants who opened the survey and provided consent, 102 completed the survey. Most drop-outs occurred after participants had read the definitions of ID and completed the first questions concerning the diagnostic procedure. One possible explanation is that the recruitment lists included practitioners who worked with children but not necessarily with the specific target population addressed by the survey. Some participants may therefore have discontinued the survey after determining that the topic was not sufficiently relevant to their professional practice. In addition, the survey comprised questions of high relevance to the target population but was relatively time-consuming, which may have further contributed to participants discontinuing the survey. As reasons for individual drop-outs were not collected, these explanations remain speculative. Nevertheless, the relatively high drop-out rate may have introduced self-selection bias, as participants who completed the survey may have been particularly interested in or familiar with the topic.

A limitation concerns the broad operationalization of the target population. Although children with ID (IQ < 70) and children with an IQ range of 70–85 were jointly included, these groups are diagnostically distinct and may differ in their clinical profiles. The present survey therefore cannot fully capture potential differences in diagnostic procedures between the two groups. To examine these differences more systematically and to further explore the lack of consensus in German-language DLD terminology (4), the relevant questions would have needed to be asked separately for children with IQ < 70 and those in the IQ 70–85 range. This was not pursued because of the additional questionnaire length. Furthermore, during the pilot study, practitioners indicated that an IQ result is often not available at the time of language assessment and that a precise distinction between these groups is not always relevant to their clinical decision-making. Nevertheless, future research should examine the two groups separately to determine whether their diagnostic procedures and clinical needs differ systematically. Additionally, the calculation of the cumulative “Experience” and “Knowledge” scores is open to criticism. Although the individual items of each score can be grouped thematically and are based on the questions from the study by Marxen et al. (68), the calculation of the scores represents more of an experimental consolidation.

### 4.6 Conclusion

This study provides insights into a previously unexplored field. It examines the approaches adopted by SLPs and other practitioners when diagnosing children with (suspected) ID, how they consider the impact of multilingualism on diagnosis for children with ID, and their self-perceptions regarding their competence in this domain.

Findings reveal an ambiguity between the application of existing, standardized procedures—the majority of which were developed for children without ID—and flexible and individual methods in order to provide a comprehensive language assessment. Practitioners navigate this discrepancy by integrating standardized tools with adaptations and qualitative assessment strategies. This may be a consequence of the lack of adequate language diagnostic materials for children with ID in German-speaking countries, as well as internationally (see chapters 1.3 and 1.4). In regard to this target group, practitioners prioritize the development of comprehensive communicative profiles in diagnosis, as opposed to the utilization of binary diagnostic decisions.

The findings in chapter 3.1.5 demonstrate that, while practitioners do not adjust their diagnostics as much for the BIF IQ range as they do for IQ < 70, they do make adjustments compared to conventional DLD diagnostics. It could therefore be helpful for SLPs in German-speaking countries if they had a clearer classification of IQ ranges in terms of terminology. For instance, if the IQ range of 70–85 were explicitly classified as DLD, as it is the case in CATALISE (2), this could serve as a clear inclusion criterion for future studies. In addition, practitioners would know to conduct their standard DLD assessments with children within this IQ-range.

In the context of multilingualism, it has been observed that for the majority of SLPs, multilingualism does not appear to be a determining factor in making different diagnostic material decisions. While the majority of practitioners do not perceive a negative impact on language development in children with ID and advise parents to speak their heritage language, a small proportion of SLPs view multilingualism as a potential negative influence on language development.

The surveyed SLPs’ self-perception of competence appears to be moderate. The results regarding potential influencing factors show that confidence in assessing DLD in children with (suspected) ID is associated with clinical experience and specific working environments, whereas no significant associations were found for knowledge acquisition through reading specialist literature or training courses.

The results of the study highlight the need for validated language assessment tools and clearer diagnostic guidelines tailored to children with ID. Consequently, additional continuing education programs for SLPs are required to help them broaden their expertise, and strengthen their professional perception of competence. A more precise consensus on terminology in German-speaking countries regarding the definition of DLD, as in CATALISE (2), could also result in a more confident and consistent approach among practitioners. Furthermore, the findings indicate that SLPs recognize the significance of holistic and individualized approaches in clinical decision-making.

## Data Availability

The raw data supporting the conclusions of this article will be made available by the authors, without undue reservation.
